# The effects of mobile-based education on nurse self-concept among nursing students: an embedded mixed methods study*

**DOI:** 10.17533/udea.iee.v41n1e15

**Published:** 2023-03-14

**Authors:** Zohreh Badiyepeymaiejahromi, Shahrzad Yektatalab, Marzieh Momennasab, Manoosh Mehrabi

**Affiliations:** 1 Nurse, Ph.D. Assistant Professor. Department of Nursing, Jahrom University of Medical Sciences, Jahrom, Iran. Email: z.badiyepeyma@gmail.com. Department of Nursing Jahrom University of Medical Sciences Jahrom Iran z.badiyepeyma@gmail.com; 2 Nurse, Ph.D. Associate Professor. Department of Nursing, Shiraz University of Medical Sciences, Shiraz, Iran. Email: yektash@sums.ac.ir. Department of Nursing Shiraz University of Medical Sciences Shiraz Iran yektash@sums.ac.ir; 3 Nurse, Ph.D. Associate Professor. Department of Nursing, Shiraz University of Medical Sciences, Shiraz, Iran. Email: momennasab@sums.ac.ir Department of Nursing Shiraz University of Medical Sciences Shiraz Iran momennasab@sums.ac.ir; 4 . Nurse, Ph.D. Assistant Professor. Department of e-Learning in Medical Sciences, Shiraz University of Medical Sciences, Shiraz, Iran. Email: mehrabi.manoosh@gmail.com. Department of e-Learning in Medical Sciences Shiraz University of Medical Sciences Shiraz Iran mehrabi.manoosh@gmail.com

**Keywords:** mobile applications, self concept, students, nursing, qualitative research, nursing education research, aplicaciones móviles, autoimagen, estudiantes de enfermería, investigación cualitativa, investigación en educación de enfermería, aplicativos móveis, autoimagen, estudantes de enfermagem, pesquisa qualitativa, pesquisa em educação de enfermagem

## Abstract

**Objective.:**

This study evaluates the effects of mobile-based education (MBE) on nurse self-concept (NSC) among nursing students.

**Methods.:**

This embedded mixed methods study was conducted in 2020-2021 in a main quantitative phase and a supplementary qualitative phase. In the quantitative phase, a quasi-experimental study with the Solomon four-group design was conducted on 117 second-year nursing students of Shiraz University of Medical Sciences, Shiraz, Iran. 70 students in the first (C1: *n*=37) and the second (C2: *n*=33) semesters of the 2020 academic year were respectively considered as the control groups, and 40 students in the first semester of the 2021 academic year considered as the experimental groups (I1: *n*=20 and I2: *n*=20). Participants in the experimental groups received NSC-related MBE through an Android application, while their counterparts in the control groups received no NSC-related MBE. Cowin’s Nurse Self-Concept Questionnaire was used to assess the NSC. In the qualitative phase, six students from the experimental groups were purposively selected and interviewed through face-to-face semi-structured interviews. Moreover, two focus group discussions were held with a six-person and a five-person group of students from the experimental groups.

**Results.:**

While the mean scores of NSC and its dimensions did not significantly change in the C1 group, the posttest mean scores of them in the E1 group were significantly greater than the corresponding pretest values (*p*<0.05), expect for the care dimension (*p*=0.586). Moreover, except for the care dimension (*p*>0.05), the posttest means scores of NSC and its other dimensions in the E1 and the E2 groups were significantly greater than the C1 and the C2 groups, respectively (*p*<0.05). Analysis of the qualitative data resulted in the generation of the main theme of multidimensional growth and development with three main categories, namely development of coping strategies, knowing professionalization strategies, and development of managerial potentials.

**Conclusion.:**

NSC-related MBE is effective in improving nursing students’ NSC.

## Resumen

**Objetivo.:**

Este estudio evalúa los efectos de la educación basada en el móvil (EBM) en el autoconcepto enfermero (ACE) entre los estudiantes de enfermería.

**Métodos.:**

Estudio de métodos mixtos realizado entre los años 2020-2021 tuvo una fase cuantitativa principal y una fase cualitativa complementaria. En la fase cuantitativa se llevó a cabo un estudio cuasi-experimental con el diseño de cuatro grupos de Salomón en los que se asignaron 117 estudiantes de segundo año de enfermería de la Universidad de Ciencias Médicas de Shiraz, Irán. Participaron en los grupos de control 70 estudiantes: 37 del primer (C1) y 33 del segundo (C2) semestres del año académico 2020 y 40 estudiantes del primer semestre de 2021 como los grupos de intervención (I1: *n*=20, e I2: *n*=20). Los participantes de los grupos de intervención recibieron EMB relacionados con el CNS a través de una aplicación Android, mientras que sus homólogos de los grupos de control no recibieron este tipo de educación. Se utilizó el Cuestionario de Autoconcepto de Enfermería (ACE) de Cowin. En la fase cualitativa, se seleccionaron intencionadamente seis estudiantes de los grupos experimentales a quienes se les realizaron entrevistas semiestructuradas cara a cara. Además, se hicieron dos grupos de discusión con un grupo de seis y otro de cinco estudiantes de los grupos experimentales.

**Resultados.:**

Mientras que las puntuaciones medias del ACE y sus dimensiones no cambiaron significativamente en el grupo C1, las puntuaciones medias pos-test de las mismas en el grupo I1 fueron significativamente mayores que los correspondientes valores pre-test (*p*<0.05), excepto para la dimensión de cuidado (*p*=0.586). Además, con excepción de la dimensión de atención (*p*>0.05), las puntuaciones medias pos-test de ACE y sus otras dimensiones en los grupos I1 y I2 fueron significativamente mayores que las de los grupos C1 y C2, respectivamente (*p*<0.05). El análisis de los datos cualitativos dio como resultado la generación del tema principal de crecimiento y desarrollo multidimensional con tres categorías principales: el desarrollo de estrategias de afrontamiento, el conocimiento de estrategias de profesionalización y el desarrollo de potenciales de gestión.

**Conclusión.:**

La intervención EBM es eficaz para mejorar el ACE de los estudiantes de enfermería.

## Resumo

**Objetivo.:**

Avaliar os efeitos da educação baseada em dispositivos móveis (EBM) no autoconceito de enfermagem (ACE) entre estudantes de enfermagem da Shiraz University of Medical Sciences, Irã.

**Métodos.:**

Estudo de métodos mistos que teve uma fase principal quantitativa e uma fase qualitativa complementar. Na fase quantitativa, foi realizado um estudo quase-experimental com o desenho de quatro grupos Solomon. 70 alunos participaram dos grupos de controle: 37 do primeiro (C1) e 33 do segundo (C2) semestre do ano letivo de 2020 e 40 alunos do primeiro semestre de 2021 como grupos de intervenção (I1: *n*=20, e I2: *n*=20). Apenas os participantes dos grupos de intervenção receberam EMBs relacionados à ACE por meio de um aplicativo Android. A escala de *Cowin* foi utilizada para avaliar o ACE. Na fase qualitativa, foram selecionados intencionalmente seis alunos dos grupos experimentais aos quais foram realizadas entrevistas semiestruturadas face a face. Além disso, foram feitos dois grupos de discussão (um de seis e outro de cinco) com alunos dos grupos experimentais.

**Resultados.:**

Enquanto as pontuações médias do ACE e suas dimensões não mudaram significativamente no grupo C1, as pontuações médias pós-teste no grupo I1 foram significativamente maiores do que os valores pré-teste correspondentes (*p*<0.05), exceto para a dimensão do cuidado (*p*=0.586). Além disso, com exceção da dimensão atenção (*p*>0.05), as pontuações médias pós-teste do ACE e de suas outras dimensões nos grupos I1 e I2 foram significativamente maiores do que nos grupos C1 e C2, respectivamente (*p*<0.05). A análise dos dados qualitativos resultou na geração do tema principal crescimento e desenvolvimento multidimensional com três categorias principais: o desenvolvimento de estratégias de enfrentamento, o conhecimento de estratégias de profissionalização e o desenvolvimento de potenciais de gestão.

**Conclusão.:**

A intervenção EBM foi eficaz em melhorar o ACE de estudantes de enfermagem.

## Introduction

Nurse self-concept (NSC) is important for the growth and development of the nursing profession because the nursing profession not only needs expertise in nursing skills but also nurses who are psychologically prepared to care for individuals.^1^ NSC is defined as the subjective experiences, perceptions, and feelings of nurses towards themselves as nurses and towards their nursing roles.^2^ NSC has six dimensions, namely general self-concept, knowledge, care, communication, staff relations, and leadership. General self-concept is positive self-feeling due to being a nurse. The knowledge dimension refers to mastery over nursing skills and theories and their use in practice. The care dimension is the perceived ability to support others. The communication dimension is the perceived ability to communicate with other healthcare professionals and patients, while the staff relations dimension refers to the perceived ability to communicate with colleagues. Finally, the leadership dimension is the perceived ability to guide the activities of the healthcare team.^1^

NSC has significant effects on their professional identity,^3^ job satisfaction, retention in the profession,^4^ academic burnout,^5^ and job burnout.^6^ But when the self-concept of nursing students is not developed in a positive direction, there are problems in transferring from education to job, inability to speak about profession, inability to successfully perform the role of nursing, and lack of communication between the person and the job.^7^ Low nursing self-concept can have a negative effect on the patient's healthcare.^8^ Furthermore, many different factors can affect NSC. For instance, a descriptive study in Turkey (2017) reported high NSC among female nurses as well as among nurses with a higher university degree, older age, and greater work experience.^9^ Moreover, factors such as the public image of nursing, environment, and professional and socio-cultural values can affect NSC.^10^

Education is a factor with potential effects on NSC.^10^^,^^11^ Education that presents positive aspects of the nursing profession can positively affect students’ NSC and retention in the profession.^9^ An experimental study found that a two-day teacher-centered educational workshop had significant positive effects on NSC and its general self-concept, care, knowledge, and communication dimensions among nursing students.^11^ Another quasi-experimental study in the United States found a wellness course effective in improving NSC and its leadership and communication dimensions among second-year nursing students.^12^ Mobile-based education (MBE) is a modern teaching method with great popularity among students.^13^ MBE does not necessitate the presence of learners in a specific place.^14^ A mixed methods study showed the effectiveness of MBE through the short message service in encouraging nursing students to remain in the profession.^15^ Limited descriptive studies have examined the NSC of nursing students with different results. For example, the mean score of NSC was reported to be 6.54 (in the possible range of 1-8) among nursing students in China (5) and 192.71 (in the possible range of 36-288) among senior nursing students in Iran.^16^

There are no data on the effects of MBE on nursing students’ NSC. Moreover, studies into the effects of education on NSC mainly used teacher-centered methods. NSC is also a context-bound concept and hence, the results of studies in one context cannot easily be generalized to other contexts. Therefore, the present study was conducted to evaluate the effects of MBE as a student-centered method on NSC among nursing students.

## Methods

This embedded mixed methods study was conducted in 2020-2021 in a main quantitative phase and a supplementary qualitative phase. 

### The quantitative phase Method

*Design.* A quasi-experimental study with the Solomon four-group design was conducted in the first and the second semesters of the 2020 academic year and the first semester of the 2021 academic year in Shiraz University of Medical Sciences, Shiraz, Iran. The Solomon design^17^ is one of the most powerful quantitative research designs which eliminates the confounding effects of the pretest on the results (Table 1). This phase aimed to evaluate the effects of MBE on NSC among nursing students.

**Table 1:** The Solomon four-group design

Posttest	Intervention	Pretest	Groups
+	+	+	Experimental 1 (E1)
+	-	+	Control 1 (C1)
+	+	-	Experimental 2 (E2)
+	-	-	Control 2 (C2)

*Study setting and participants.* This study was conducted in Hazrat-e Fatimah Faculty of Nursing and Midwifery of Shiraz University of Medical Sciences, Shiraz, Iran. This university is the largest university in the south of Iran and one of the leading universities in this country. The faculty admits BSN students in both the first and the second semesters of each academic year. Study participants were second-year BSN nursing students in the 2020 (n=74) and 2021(n=43) academic years. Only second-year students were included because they may have low NSC.^18^ Also, due to the fact that some time has passed since the second-year nursing students attended the nursing faculty, they are familiar with nursing courses and they have one semester of experience in the clinical environment, the researchers have chosen them to conduct the intervention. All enrolled second-year BSN students were recruited for participation. Participation was voluntary; thus, students who were reluctant to participate and those who did not complete the instrument in its entirety were excluded.

*Instrument.* Cowin’s Nurse Self-Concept Questionnaire (NSCQ) was used for NSC assessment. This questionnaire has 36 items in six six-item dimensions, namely general self-concept, knowledge, care, communication, staff relations, and leadership. All items are positively worded and are scored on a 1-8 Likert scale, resulting in a total score of 36-288 with higher scores representing greater NSC. Previous studies reported the acceptable validity and reliability of the Turkish,^19^ Chinese,^20^ and Persian^21^ versions of this questionnaire. Participants completed the Persian version of this questionnaire through the self-report method. According to the Persian study, Cronbach's alpha of the total questionnaire was 0.95, and in the dimensions of general self-concept (0.96), care (0.91), knowledge (0.91), communication (0.94), staff relations (0.92), and leadership (0.94) were estimated.^22^ The reliability of the questionnaire was also investigated in a pilot study on nursing students; after completing the questionnaire by 30 nursing students except for students in the study groups, Cronbach's alpha of total questionnaire (0.96) and different dimensions of general self-concept (0.96), care (0.88), knowledge (0.88), communication (0.92), staff relations (0.91), and leadership (0.94) were confirmed.

*Intervention.* Students in the first and the second semesters of the 2020 academic year (*n*=39 and 35, respectively) were considered as the control 1 (C1) and the control 2 (C2) groups, respectively. Participants in these groups received no MBE or NSC-related intervention. Students in the C1 group were assessed for NSC both at the pretest and the posttest while their counterparts in the C2 group were assessed for NSC only at posttest. Second-year nursing students in the first semester of the 2021 academic year were randomly allocated to two experimental groups, namely the E1 (*n*=22) and the E2 (*n*=21) groups. MBE was provided to all students in these two experimental groups in a similar way. Students in the E1 group were assessed for NSC both at the pretest and the posttest while students in the E2 group were assessed for NSC only at the posttest. Posttest in both experimental groups was performed one month after providing them with the educational program.

The intervention of the study was a multimedia MBE program which was provided through an Android application. Educational materials were provided in the three main categories of professional values (namely professional ethics and autonomy), life skills (namely self-esteem, stress and adaptation, critical thinking, and communication skills), and managerial skills (namely teamwork, self-regulation, and leadership). These three categories were developed based on a qualitative study into the factors affecting nursing students’ NSC.^23^ The content of the program was developed using relevant articles and textbooks and was approved by five nursing instructors from Shiraz University of Medical Sciences, Shiraz, Iran. Educational audio and video clips of the program were produced using the Camtasia program. With an appointment, the first author of the study held a twenty-minute session with participants in the experimental groups and provided them with the Android application of the study intervention through the SHAREit application. Participants installed the application on their tablets or mobile phones and then, the first author provided them with explanations about how to use the application and its different parts. The first author supervised and supported them through the WhatsApp application and telephone contacts. Accordingly, all participants in the experimental groups were added to a WhatsApp group managed by the first author, where they could text and interact with each other and the group manager about the use of technology and discussion of teaching topics. There was no time limit on student interaction and text on WhatsApp group. The final discussion and conclusion were done at the end of the study of each main category of MBE. The students completed MBE in eight weeks.

*Data analysis.* Data were analyzed through the SPSS software (v. 21.0). The Kolmogorov-Smirnov test showed that all variables of the study had normal distribution (*p*<0.05). Therefore, the independent-sample *t*, paired-sample *t*, and Chi-square tests as well as the one-way analysis of variance were used for data analysis. The data were presented using the measures of descriptive statistics, including frequency, mean, and standard deviation.

### The qualitative phase method

*Design.* A descriptive qualitative study was conducted in the first semester of the 2021academic year through the conventional content analysis approach to explore participants’ experiences of the study intervention.

*Participants and data collection.* Participants were six students purposively recruited from the E1 (*n*=3) and E2 (*n*=3) groups. They were selected from those students who had the smallest and the biggest pretest-posttest changes in the mean score of NSC. Personal semi-structured interviews were held with these six students. Besides, two focus group discussions were held with a group of six students and another group of five students. At this stage, no new data was extracted and we reached data saturation. The participants of the focus groups were different from the personal interviews. Interviews and focus groups were started using these open-ended questions, “What were the effects of the educational program on your attitude towards the nursing profession?” “What was your perception of yourself as a nurse after the program?” and “May you please provide examples of your behaviors which can show the effects of the program?”. Pointed questions were also used to collect more detailed data. Interviews were held in a room in the first author’s workplace and focus group discussions were held in a conference room in the study setting. Face to face interviews and focus group discussions lasted 30-60 minutes, were digitally recorded, and transcribed verbatim. The transcripts were imported to the MAXQDA 2007 software for data management and analysis.

*Data analysis.* Data were analyzed through Graneheim and Lundman five-step conventional content analysis approach. The first researcher independently analyzed the data. Accordingly, each transcript of the data was read several times by the first researcher in order to grasp its main ideas. Then, meaning units were determined and coded. Generated codes were categorized according to similarities and finally, the latent content of the data was identified and presented in the form of main categories and themes.^24^

*Trustworthiness.* Lincoln and Guba’s criteria were used to establish trustworthiness.^25^ Credibility was ensured through prolonged engagement with the study subject matter, member checking, peer checking, and sampling with maximum variation in terms of participants’ gender and change in the mean score of NSC. Bracketing, peer checking, and constant comparison of the data helped establish dependability. Confirmability was ensured through keeping all documents related to the study and performing external peer checking. Moreover, clear explanations were provided about participants’ characteristics, study setting, sampling, data collection, and findings in order to ensure transferability.

Ethical considerations. The Ethics Committee of Shiraz University of Medical Sciences approved the study (IR.SUMS.REC.1397.215). An opening session was held with participants in order to inform them about the study, the voluntariness of participation, freedom to withdraw from the study, and confidentiality of the study data. Students who participate in the study were not the researchers' students, they have no coercion for participation and ensure that refusal to participate or remain in the study would never affect their final evaluation scores. Participants signed the written informed consent form of the study. At the end of the study, the Android application of the study intervention was provided to all participants in the control groups.

**Figure:**
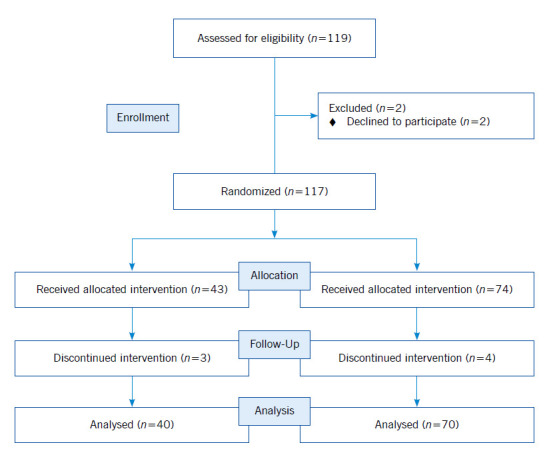

## Results

### The findings of the quantitative phase

Two students in C1, two students in C2, two students in E1, and one student in E2 were excluded due to unwillingness to participate or continue in the study. Finally, the data of 37 students in C1, 33 students in C2, 20 students in E1, and 20 students in E2 were analyzed. The means of participants’ age and grade point average were 21.38±2.92 years and 15.27±1.99, respectively. The Iranian grades point average ranges from 0 to 20. The passing grade is 10. Grades 17 or more are considered excellent, grades 14 to 16.99 are considered good and grades 10 to 13.99 are considered acceptable. Students having 9.99 or less fail.

50.9% of participants were female. Most participants were single (88.2%) and had no clinical work experience (90.0%). Groups did not differ significantly with respect to participants' age, grade point average, gender, marital status, and clinical work experience (*p*<0.05; Table 2).

**Table 2:** Among-group comparisons respecting the characteristics of the participants of the quantitative phase

***p*-value**	C2 (*n*=33)	C1 (*n*=37)	E2 (*n*=20)	E1 (*n*=20)	Groups Characteristics
0.977*	21.48±3.68	21.45±2.80	21.30±1.97	21.15±2.43	Age (Years); Mean±SD
0.969*	15.22±1.57	15.38±2.78	15.34±1.77	15.11±1.52	Grade point; Mean±SD
					Gender; *n* (%)
0.633^	15 (40.5)	17 (51.5)	11 (55.0)	11 (55.0)	Male
	22 (59.5)	16 (48.5)	9 (45.0)	9 (45.0)	Female
					Marital status; *n* (%)
0.248^	32 (86.5)	27 (81.8)	20 (100.0)	18 (90.0)	Single
	5 (13.5)	6 (18.2)	0 (0.0)	2 (10.0)	Married
0.185^					Clinical work experience; *n* (%)
	18 (90.0)	18 (90.0)	18 (90.0)	18 (90.0)	No
	20 (100.0)	20 (100.0)	20 (100.0)	20 (100.0)	Yes

*: The results of the one-way analysis of variance; ^: The results of the Chi-square test

Control 1 (C1), Control 2 (C2), Experimental 1 (E1), Experimental 2 (E2)

Table 3 showed no significant difference between the E1 and the C1 groups respecting the pretest mean scores of NSC and all its dimensions (*p*>0.05). Table 3 indicated that in the E1 group, the post-test means scores of NSC and all its dimensions except for the care dimension were significantly greater than their corresponding pretest values (*p*<0.05). However, the mean scores of NSC and its dimensions did not significantly change in the C1 group (*p*>0.05). It revealed that except for the mean score of the care dimension, the differences between the E1 and the C1 groups and between the E2 and the C2 groups respecting the posttest mean scores of the dimensions of NSC were statistically significant (*p*<0.05). Table 3 also showed that the differences between the E1 and the E2 groups and between the C1 and the C2 groups respecting the posttest mean scores of NSC and its dimensions were not statistically significant (*p*>0.05). These findings confirmed that the pretest mean scores had no significant effects on the corresponding posttest mean scores.

**Table 3:** Within-group and pairwise between-group comparisons respecting the mean scores of NSC and its dimensions

Outcomes	Time/Group	E1	C1	E2	C2	*p*-value*	
Total	Pretest	215.95±42.62	222.00±35.70	-	-	0.408	
	Posttest	239.90±20.29	214.24±39.70	235.65±14.92	214.43±36.66	E1 vs. E2: 0.455	C1 vs. C2: 0.983
	p value^	0.001	0.335	-	-	E1 vs. C1: 0.003	E2 vs. C2: 0.003
General self-concept	Pretest	31.70±10.66	33.96±9.34	-	-	0.420	
	Posttest	38.85±5.65	33.78±8.54	36.25±5.04	32.59±8.54	E1 vs. E2: 0.133	C1 vs. C2: 0.562
	p value^	0.001	0.917	-	-	E1 vs. C1: 0.012	E2 vs. C2: 0.047
Care	Pretest	38.20±7.95	36.93±5.97	-	-	0.515	
	Posttest	37.55±3.79	35.45±6.71	38.45±3.36	37.59±6.86	E1 vs. E2: 0.432	C1 vs. C2: 0.193
	p value^	0.586	0.246	-	-	E1 vs. C1: 0.153	E2 vs. C2: 0.531
Knowledge	Pretest	38.00±7.67	38.78±7.82	-	-	0.722	
	Posttest	41.70±2.99	37.54±7.29	39.95±3.10	36.08±6.92	E1 vs. E2: 0.077	C1 vs. C2: 0.392
	p value^	0.014	0.410	-	-	E1 vs. C1: 0.006	E2 vs. C2: 0.005
Staff relations	Pretest	36.35±6.89	37.84±6.11	-	-	0.414	
	Posttest	41.45±3.74	35.81±7.56	39.85±2.88	36.16±7.17	E1 vs. E2: 0.139	C1 vs. C2: 0.846
	p value^	0.000	0.128	-	-	E1 vs. C1: 0.001	E2 vs. C2: 0.008
Communication	Pretest	37.10±7.01	38.90±6.63	-	-	0.351	
	Posttest	41.75±3.05	37.36±7.37	40.95±4.19	37.24±6.54	E1 vs. E2: 0.495	C1 vs. C2: 0.943
	p value^	0.001	0.262	-	-	E1 vs. C1: 0.004	E2 vs. C2: 0.012
Leadership	Pretest	31.55±12.14	35.54±9.41	-	-	0.186	
	Posttest	39.60±4.58	34.27±9.11	40.20±4.60	34.75±8.15	E1 vs. E2: 0.682	C1 vs. C2: 0.815
	p value^	0.001	0.521	-	-	E1 vs. C1: 0.007	E2 vs. C2: 0.002

*: The results of the independent-sample *t* test; ^: The results of the paired-sample *t* test

Control 1 (C1), Control 2 (C2), Experimental 1 (E1), Experimental 2 (E2)

### The results of the qualitative phase

The participants ranged in age from 19-25 years (9 women and 8 men).

Participants’ main perception of the effects of NSC-related MBE was *Multidimensional Growth and Development* that refers to the development of personal and professional knowledge and abilities which improve students’ NSC and pave their way for professional development. The three main categories of this theme were development of coping strategies, knowing professionalization strategies, and development of managerial potentials.

*Development of coping strategies.* Coping strategies are a set of cognitive and behavioral efforts for managing and controlling stressful situations. According to the participants, many different stressors affect nursing students. The ineffective management of these stressors can cause problems such as poor NSC and college dropout. Participants noted that the study intervention improved their ability to cope with and manage their stress: *In the previous term, we had the clinical course of the Fundamentals of Nursing. Sometimes, I experienced stress and decided to drop out. But, now I know how to manage my stress and feel lower stress. I see that the educational program has had positive effects (P. 8).* Participants also noted that they had problems in finding appropriate information for managing their problems and confirmed that the study intervention improved their abilities to find appropriate information and to manage their problems: *I learned that we should not be routine-oriented persons; rather, we should think about the possible solutions to problems, study or ask experienced persons about them, and use the internet or any other resources in order to find the best solutions which can prevent many other problems (P. 4).* Participants’ experiences also showed the positive effects of the study intervention on their NSC and their image of nursing. They noted that improvement in NSC and professional image is a type of internal strengthening. Positive changes in students’ professional image helped them value themselves and their profession and more effectively cope with problems. Participants noted that the provision of such NSC-related educations to students at university can significantly improve their NSC as nurses: *Participation in this program caused me enjoy to be a nurse. I tell myself that I can be one of the best nurses in a near future (P. 13).*

*Recognition of professionalization strategies.* Participants also noted that participation in the NSC-related MBE program helped them recognize professionalization strategies, including development of communication skills, promotion of teamwork, dynamic learning, and NSC improvement. They considered effective communication as a key requirement of professional role performance in nursing and noted that they needed to learn communication skills in order to establish effective interpersonal communications: *Previously, I knew almost about 30% of communication skills; but now I know that communication skills have many different aspects, know many things about them, and will definitely use them (P. 11).* Participants believed that besides professional knowledge, nursing staff should learn communication and teamwork skills. They noted that the study intervention gave them a positive attitude towards teamwork and helped them appropriately learn many things about teamwork: *The program appropriately taught us the principles of teamwork and showed us how to do teamwork and establish interpersonal communication in different clinical situations (P. 7).* Participants also referred to the positive effects of the study intervention on their motivation for developing their professional knowledge and clinical skills. They noted that after the intervention, they not only did not escape from the lessons, but also attempted to learn modern teaching methods. Their comments also showed that they were interested in the process of MBE and considered it beneficial and applicable: *Now, I know that I need to study more. When I’m idle, I use internet or study the terminology book. I attempt to know more about illnesses in order to develop my knowledge before attending our clinical courses (P. 5); MBE is very good. It is always accessible and easy to use (P. 13).*

*Development of managerial potentials.* The third main category of the multidimensional growth and development main theme was the development of students’ managerial skills. Participants noted that despite the importance of management and leadership, they had received little education, if any, in these areas during the first years of their university education and hence, did not have a good understanding about nurses’ managerial roles. They highlighted that the study intervention had positive effects on their understanding of the principles of nursing management: *Now, I understand the tasks of a manager and know that he/she organizes the affairs, fairly assigns tasks to different staff, supervises their task performance, and knows the responsibilities of each staff (P. 5).* Participants also highlighted the necessity of providing students with education about professional management and leadership in order to improve their managerial and leadership skills. They also acknowledged that performing any professional task or role of nursing necessitates the use of different managerial and leadership skills: *The management part of the program was very good because each nurse needs to have managerial power. Even a simple nurse who is not a head nurse should know these things because this knowledge enables him/her to plan for patient care or promote collaboration among healthcare providers, from service workforces to physicians (P. 6).*

### Integration of the results of the quantitative and the qualitative phases

The results of both quantitative and qualitative phases indicated the effectiveness of the NSC-related MBE program on nursing students’ NSC and its dimensions, except for its care dimension. 

## Discussion

This study evaluated the effects of MBE on NSC among nursing students. The results of the quantitative phase of the study showed significant improvement in the mean scores of NSC and its general self-concept, knowledge, communication, staff relations, and leadership dimensions. The results of the qualitative phase also confirmed students’ personal and professional development after the intervention. According to the participants, the study intervention gave them a positive attitude towards nursing and increased their awareness of the necessary requirements and skills for becoming a nurse.

We could not find any study into the effects of MBE on NSC for the sake of comparison. However, a qualitative study showed that students’ interviews with different healthcare providers provided them with opportunities to develop their professional experiences, perceptions, interactions, and NSC.^26^ Another quantitative study found that peer-mentoring by fourth-year nursing students provided sophomore students with the opportunities to exercise managerial and leadership skills and work as a member of healthcare team and thereby, positively affected their NSC.^27^ Similarly, a quantitative study found that wellness course had positive effects on NSC among second-year nursing students.^12^ Moreover, a quantitative study showed that education through a workshop on self, self-concept, NSC, communication, management and leadership, nursing care, and professional ethics had positive effects on NSC among fourth-year students.^11^ Another quantitative study also found that education and orientation sessions on the status and values of nursing, definition, and characteristics of a profession, nurses’ professional roles, global attitude towards nursing, personal responsibilities towards the profession, communication skills, strategies to be a good nurse, and techniques for improving NSC had significant positive effects on students’ NSC.^28^ All these findings imply that sharing professional experiences with students, preparing them for professional practice, providing them with education, and introducing nursing as an independent profession positively affect their professional development and maturity and thereby, can improve their NSC. Such strategies are particularly important for first- and second-year students who may have feelings of isolation and rejection and may decide on quitting the profession.^15^

In line with our findings, a former mixed method study found that MBE through supportive and encouraging text messages encouraged nursing students to remain in the profession and improve their sense of belonging to the university.^15^ Another quantitative study found that a mobile phone application on self-awareness significantly improved students’ awareness of how they spent their time.^29^ These findings confirm the positive effects of MBE and the necessity of using mobile phones for providing education to students.

Findings also revealed that the MBE intervention of the study had no significant effects on the care dimension of NSC. In the qualitative phase, participants also did not report any change in their clinical skills. In line with these findings, a former quantitative study showed the ineffectiveness of a wellness course in significantly improving the care dimension of NSC among nursing students.^12^ The insignificant change of the care dimension of NSC after the study intervention may be due to the fact that care is the practical aspect of the nursing profession and its improvement needs practical training, while our intervention mainly focused on NSC promotion and stress management and included no practical training. Moreover, our participants were second-year students who were at the first stages of their clinical education and had no considerable clinical skills. Contrary to our findings, two former quantitative studies reported that NSC-related education significantly improved the mean score of the care dimension of NSC.^11^^,^^27^ This contradiction may be due to the fact that those two studies directly addressed the care dimension and were conducted on fourth-year nursing students who usually have greater clinical skills. Therefore, practical NSC-related education, particularly about the care dimension of NSC, is recommended for improving all aspects of NSC among nursing students.

Participants in the qualitative phase of the study also highlighted that the MBE intervention of the study helped them develop a positive attitude towards MBE. In agreement with this finding, a systematic review and meta-analysis concluded that MBE is effective in significantly improving nursing students’ attitudes towards learning and developing their knowledge, skills, and self-confidence.^30^ The positive effects of MBE on students’ attitudes towards MBE are mostly due to the applicability and perceived simplicity and usefulness of MBE.^31^^,^^32^ Moreover, the different options of mobile phones provide the opportunity to provide students with more interesting and more diverse educational materials which can, in turn, promote their motivation for learning and improve their attitudes.^33^

One of the strengths of the study was its mixed methods design which helped produce more reliable results.^34^ Moreover, the use of the Solomon four-group design in the quantitative phase helped remove the confounding effects of the pretest. Nonetheless, we could not assess the long-term effects of the study intervention due to time limitation. Studies with longer courses and longer follow-up assessment durations are recommended to assess the long-term effects of MBE on NSC. As few interventional studies have been conducted for NSC promotion, future studies are recommended to use interventional designs to assess the effects of modern teaching methods such as MBE on nursing students’ NSC. In addition, if studies were carried out comparing traditional teaching methods of these concepts to MBE to determine which methods have greater effectiveness in student learning would add to the science of nursing education. Other limitations include the cross-sectional nature of the study, the single study setting, small sample of students, and completion of questionnaires as self-report.

The results of this study can be used as a basis for future quantitative and qualitative studies into NSC. The NSC-related Android application developed in the present study can be provided to nursing students to improve their NSC and professional skills such as communication and stress management skills. Also, nurse educators could apply mobile-based applications in the future to support their students.

## Conclusion

The findings of this study conceptualized the impact of NSC-related MBE in the form of an educational intervention for improving NSC. For the first time in a mixed method research, it showed quantitative and qualitative evidence of the effect of this intervention on improving the students' attitudes towards nursing, and positively affecting their academic and professional life**.** The findings of the present study provide a detailed insight into the effects of a mobile-based student-centered teaching method for NSC promotion. NSC-related MBE is recommended for providing nursing students with education about different aspects of nursing and improving their NSC.
